# Prevalence and sociodemographic correlates of stunting, underweight, and overweight among Palestinian school adolescents (13-15 years) in two major governorates in the West Bank

**DOI:** 10.1186/1471-2458-9-485

**Published:** 2009-12-23

**Authors:** Nahed Mikki, Hanan F Abdul-Rahim, Faisal Awartani, Gerd Holmboe-Ottesen

**Affiliations:** 1 Section of Preventive Medicine and Epidemiology, Institute of General Practice and Community Medicine, University of Oslo, Oslo, Norway; 2 Institute of Community and Public Health, Birzeit University, Ramallah, Occupied Palestinian territory; 3 International Affairs Department, Qatar University, Doha, Qatar; 4 Alpha International for Research, Polling and Informatics, Ramallah, Occupied Palestinian territory

## Abstract

**Background:**

There is little information about height and weight status of Palestinian adolescents. The objective of this paper was to assess the prevalence of stunting, underweight, and overweight/obesity among Palestinian school adolescents (13-15 years) and associated sociodemographic factors in 2 major governorates in the West Bank.

**Methods:**

A Cross-sectional survey was conducted in 2005 comprising 1942 students in 65 schools in Ramallah and Hebron governorates. Data was collected through self-administered questionnaires from students and parents. Weights and heights were measured. Overweight and obesity were assessed using the 2000 Centers for Disease Control and Prevention (CDC) reference and the International Obesity Task Force (IOTF) criteria. Stunting and underweight were assessed using the 2000 CDC reference.

**Results:**

Overweight/obesity was more prevalent in Ramallah than in Hebron and affected more girls than boys. Using the 2000 CDC reference, the prevalence of overweight and obesity in Ramallah among boys was 9.6% and 8.2%, respectively versus 15.6% and 6.0% among girls (P < 0.01). In Hebron, the corresponding figures were 8.5% and 4.9% for boys and 13.5% and 3.4% for girls (P < 0.01). Using the IOTF criteria, the prevalence of overweight and obesity among boys in Ramallah was 13.3% and 5.2%, respectively versus 18.9% and 3.3% for girls. The prevalence of overweight and obesity among boys in Hebron was 10.9% and 2.2%, respectively versus 14.9% and 2.0% for girls. Overweight/obesity was associated with high standard of living (STL) among boys and with the onset of puberty among girls. More boys were underweight than girls, and the prevalence was higher in Hebron (12.9% and 6.0% in boys and girls, respectively (P < 0.01)) than in Ramallah (9.7% and 3.1% in boys and girls, respectively (p < 0.01)). The prevalence of stunting was similar in both governorates, and was higher among boys (9.2% and 9.4% in Ramallah and Hebron, respectively) than among girls (5.9% and 4.2% in Ramallah and Hebron, respectively). Stunting was negatively associated with father's education among boys and with urban residence, medium STL and onset of puberty among girls.

**Conclusion:**

Under- and overnutrition co-exist among Palestinian adolescents, with differences between sexes. Region, residence, STL, and onset of puberty were associated factors.

## Background

Malnutrition during adolescence has important short and long-term health implications. Adolescent obesity has been associated with adverse cardiovascular [1], metabolic [2] and psychological outcomes [3]. Tracking of obesity from adolescence to adulthood has been shown to be high [4], and obesity in adulthood is associated with chronic diseases and increased mortality [5]. At the same time, undernutrition during childhood has been linked to higher risk of obesity and chronic diseases in adulthood [6].

The rise in obesity and chronic diseases worldwide has been linked to urbanization, which entails nutrition transition and sedentary lifestyle [7]. Compared to western societies, Middle Eastern countries experienced a later but faster-paced nutrition transition [8]. Palestinian society has also been undergoing nutrition transition [9], where studies have shown nutrition-related chronic diseases and their risk factors such as heart diseases, hypertension, diabetes, cancer, smoking, sedentary lifestyle and obesity to be prevalent among Palestinians [9]. One study among adults estimated combined prevalence of overweight and obesity to be 58.7% among men and 71.3% among women [9]. At the same time, stunting which is associated with chronic undernutrition, has been increasing among Palestinian children younger than 5 years of age from 7.2% in 1996 to 10.2% in 2006 [10].

Studying various aspects of the nutritional status of Palestinian adolescents (10-19 years) is important as they represent about one-quarter of the Palestinian population [11]. Studies exploring nutrition-related aspects of Palestinian adolescents in the West Bank are few. One national study which was part of the Health Behaviour in School aged Children Survey (HBSC) was conducted in the West Bank and the Gaza Strip in 2003/2004. In that survey, prevalence of overweight/obesity (using International Obesity Task Force (IOTF) criteria) was estimated at 20.4% for boys and 13.0% for girls [12]. However, calculation of body mass index (BMI) in that survey was based on self reported weights and heights rather than on actual measurements. Moreover, undernutrition (stunting and underweight) was not assessed. To our knowledge, no population based studies have reported actual measured anthropometric data for underweight, overweight, obesity and stunting among adolescents in the West Bank. Therefore, there is a need to conduct such studies to be able to identify the vulnerable groups and plan proper intervention programs.

The West Bank in the occupied Palestinian territory (oPt) is an area of 5655 square Km with 2 350 583 inhabitants and is composed of 11 governorates (governorate is official administrative division of the oPt) [11]. This study was conducted in two main governorates in the West Bank (Ramallah and Hebron governorates) chosen for their contrasting demographic and lifestyle features and for their importance in terms of economic status and population size. Ramallah governorate is located in the middle of the West Bank with 279,730 inhabitants in 2007 [11]. It has 6 urban and 69 rural localities with 34.1% and 59.5% of the population, respectively [13]. Ramallah city is a cultural, political, and administrative center of the Palestinian Authority. Hebron, which is the largest governorate in terms of population size, is located in the southern part with 552,164 inhabitants in 2007 [11]. It has 10 urban and 144 rural localities, with 67.0% and 30.2% of the population, respectively [13]. In a comparison of demographic and lifestyle characteristics such as fertility levels, age of women at marriage, education levels, types of employment, and the availability of modern household amenities, Ramallah and Hebron cities represented two distinctive modes of urban life with Ramallah being more modernized and having a higher standard of living [14]. Corresponding rural areas showed similar pattern with those of Ramallah having better indicators than those of Hebron. The differences between the cities and their rural areas in these indicators were more pronounced in Ramallah than in Hebron [14].

The objective of this article was to assess the prevalence of stunting, underweight, overweight, and obesity using actual measured weights and heights among school-based adolescents (13-15 years) in 2 major governorates (Ramallah and Hebron) in the West Bank in the oPt. It also aimed to study the associations between these anthropometric measurements and selected sociodemographic characteristics such as residential factors (region, urban-rural), age, gender, educational level of the parents, aspects of household economic situation, and food availability.

## Methods

### Study Sample

A list of all the students attending the 8^th ^and 9^th ^grades in 2004-2005 was provided by the Palestinian Ministry of Education and Higher Education (which included number of students per classroom). The school enrollment rate for Palestinian children in the West Bank in 2007/2008 was 84.5% in the basic education stage (1^st ^to the 9^th ^grade) [15]. The total number of 8^th ^and 9^th ^grade students was 13011 in Ramallah and 26681 in Hebron. The classes were divided into nine strata using school gender category (single sex-boy, single sex-girl and co-educational) and school ownership type (public, United Nation Relief and Works Agency (UNRWA), or private). The sample was selected using single stage probability proportional-to-size sampling procedure from each of the nine strata, using the class as the primary sampling unit.

A sample size of 1000 students in each governorate was set to have a maximum margin of error of 2.9% in the prevalence estimates of overweight/obesity using the following formula:

where p is the proportion of overweight/obesity (in this case 18%, based on a similar study from the North Gaza strip) [16]; DE is the design effect that is estimated at 1.5 based on previous studies in schools; the value 1.96, assures a confidence level of 95%; and E is the expected maximum margin of error.

As the average class size was about 31 students in Ramallah and 34 students in Hebron, 65 classes were selected: 34 in Ramallah and 31 in Hebron. All students in the chosen classes were invited to participate in the study.

### Data Collection

The cross-sectional survey was conducted between March and May 2005. Self administered questionnaires were used to collect information from students and parents. The researcher and trained field workers gave students standardized instructions on filling in the questionnaires, which were completed in the classroom in 1.5-2 hours. Field workers were present during administering the questionnaires to clarify questions when needed. The students' questionnaire included questions about age, residence, household amenities and onset of puberty. Seven core questions in the students' questionnaire were adapted from the World Health Organization (WHO)-questionnaire on Health Behaviour in School-aged children (HBSC) [17]; these were: month and year of birth, onset of puberty, ownership of car, computer and Internet connection at home. The parents' questionnaire included household information such as family size, parents' education, and indicators of economic situation such as the ability to pay bills, cover family needs and having any debts. The parents' questionnaire was taken home by students and returned to school the following day. The students' and parents' questionnaires were piloted and adjusted before the survey. The students' questionnaire was tested for reliability (one week test-retest) on a different sample of 115 students in the same age group.

### Measures

#### Anthropometric measures

Students' weights were measured in light clothes without shoes to the nearest 0.1 kg using a portable scale (Seca 780/783, Hamburg, Germany). The scales were calibrated daily at the study site with a known weight. Heights were measured to the nearest 0.1 cm using a portable stadiometer (Seca 220) mounted on the scale. Students stood up straight in bare feet, with heels, buttocks and back touching the stadiometer. Measurements were taken once and recorded by one trained field worker during the early morning classes before the school recess for all students. Intensive training was conducted before the field work to ensure reliability of the measurements and decrease inter- and intra field workers' errors. BMI was calculated as weight in kilograms divided by height in meters squared.

For the purpose of regional comparisons, BMI was categorized based on age-and sex-specific cut-off values of the 2000 Centers for Disease Control and Prevention (CDC) growth charts. The categories were underweight (< 5th percentile), normal weight (5th to 85th percentile), overweight (85th to 95th percentile), and obese (> 95^th ^percentile) [18].

For the purpose of international comparisons, BMI was also categorized using the IOTF criteria. Overweight and obesity were defined as BMI cut offs corresponding to adult cut-offs of overweight and obesity (BMI of 25 and 30 kg/m^2^, respectively) [19].

Stunting was defined as height-for-age below the 3rd percentile [18].

### Sociodemographic factors

**Age of the students **was obtained from the date of birth as reported by the students. Three age groups were constructed: age group 13: 13 years 0 months to 13 years 12 months, age group 14: 14 years 0 months to 14 years 12 months, and age group 15: 15 years 0 months to 15 years 12 months.

**Urban/rural classification **was based on the Palestinian Central Bureau of Statistics (PCBS) classification which is based on services and population size [13].

**Father's educational level **was divided into 3 categories as follows: "Low": illiteracy, less than secondary school education, "Medium": secondary school education" and "High": college, university or higher.

**Mother's educational level **was constructed in the same way as the father's, but because of few subjects in the high educational category, the last two categories were collapsed to one, that is, medium/high.

**The household standard of living (STL) index **was based on household possessions. Ownership of central heating, family car, family mobile phone, personal mobile phone, indoor bathroom, water pipes, refrigerator, full automatic washing machine, color TV, satellite, video, computer, dish washer, microwave, vacuum cleaner and internet connection were summed; each item was given a value of 1. Three categories were constructed: 'low': 0-6, 'medium': 7-10, 'high':11-16. The 1 week test-retest of the ownership of these amenities showed consistent answers ranging between 86.1% and 99.1%.

**The present household economic situation index **was constructed based on 4 questions from the parents' questionnaire using a 5-point Likert Scale where 1 represented the best situation and 5 the worst. These questions were: "Do you have enough money to cover family needs now?", "At the present time, do you have any debts?", "At the present time, to what extent do you have to postpone paying bills?", and "During the past school year, did you have to borrow money to cover family needs?" A total index was formulated by adding up these values. Three categories were constructed: 'worse off': 14-20; 'medium': 7-13; 'better off': < 7. Spearman correlation between this index and STL was 0.42 (p < 0.001).

**Food availability **was assessed based on the following question from the parents' questionnaire: "Is there enough food for all family members on daily basis now?" Food was considered available if the family had enough food for all members "most of the time" or "always."

### Onset of puberty

Onset of puberty was assessed by the question "have you reached puberty i.e. "have you had your first period?" for girls and "have you noticed deepening of your voice?" for boys. The 1 week test-retest for the onset of puberty showed consist answers in 92.3% in boys and 95.2% in girls.

### Statistical analysis

EPI 2002 (Center for Disease Control and Prevention, Atlanta, Georgia) was used to calculate BMI, BMI and height percentiles. All other statistical analyses were done using Stata 10.0 (Stata Corporation, College Station, TX) and were adjusted for cluster sampling design. The sample was weighed according to sample and population size (inverse of sampling probability) in each grade and in each governorate. χ^2 ^tests were used to compare frequencies and t-tests were used to compare means. A significance level of 0.05 was used for all statistical analyses. Separate analyses were run for boys and girls in each governorate. Multivariate logistic regression analysis was used to model the association between the outcome variables (overweight/obesity), (underweight) and (stunting) and selected sociodemographic characteristics. As no interactions were found between region (Ramallah/Hebron) and the rest of the independent variables in any of the models, regression analysis was done for both regions together.

Age, region and variables with *p *< 0.05 in the bivariate analysis tables for each gender in any of the two governorates or in both of them combined were included in the regression models. Colinearity and plausible interactions between variables in the model were investigated.

### Ethical considerations

Informed consent was obtained from parents, students and school principals with standard assurances of confidentiality. They were informed that answering the anonymous questionnaire was voluntary. Moreover, the class teacher was not present while administering the questionnaires to assure confidentiality. The study was a collaboration between the Institute of General Practice and Community Medicine at the University of Oslo and the Institute of Community and Public Health at Birzeit University. The protocol was approved by both institutes. The study was also approved by the Palestinian Ministry of Education and Higher Education, by UNRWA Office of Education and by Regional Ethical Committee of Norway.

## Results

### Sample characteristics and response rates

Two schools in Ramallah governorate refused to participate in the study and were replaced by schools from the same stratum of the sampling frame. In total, 2170 students in 65 classes were invited to participate, while 2032 (93.6%) actually participated in the study. Reasons for non-participation included: absence on the day of the survey (71) and refusal from parents or students or both (67). The analysis presented in this paper excluded 90 students being younger or older than the defined age groups of 13, 14 and 15 years. The final sample thus consisted of 1942 students (95.6% of those participating). In total, 1863 (95.9%) parents completed their questionnaires. No significant differences were found between responding and non-responding parents (n = 79) in terms of STL, residence (urban versus. rural), age groups or sex of students. However, 75.9% of non-responding parents were from Hebron (p < 0.05).

Table 1 shows that girls were slightly over-represented in the sample and that the fathers of the students were more likely to have a higher education than the mothers in both governorates. The proportion of 8^th ^grade students in the sample of Ramallah governorate was higher than that in the population, but that was not the case in Hebron (data not shown). More adolescents were in the 13 and 14 years age groups compared to the number in the 15 years age group in both governorates. In Hebron, mean family size was higher and mean STL was lower compared to Ramallah. Adolescents in Hebron were more likely to be living in urban areas (67.8% in Hebron versus 41.0% in Ramallah). Hebron had a higher proportion of students coming from households with worse off present household economic situation index than Ramallah.

**Table 1:** Sociodemographic characteristics of the study sample

	Ramallah governorate	Hebron governorate	
Sociodemographic factors	n = 935	n = 1007	P^a^
	% (n)	% (n)	
**Gender**			
Boys	46.7 (437)	45.5 (458)	
Girls	53.3 (498)	54.5 (549)	0.919
**Location of residence**			
Urban	41.0 (373)	67.8 (663)	
Rural	59.0 (537)	32.2 (315)	0.032
**Age in years**			
13	41.4 (387)	32.9 (331)	
14	44.5 (416)	48.0 (484)	
15	14.1 (132)	19.1 (192)	0.257
**Mother's education**			
Less than secondary school	72.7 (662)	77.2 (729)	
Secondary school/college or university or higher	27.3 (249)	22.8 (215)	0.322
**Father's education**			
Less than secondary school	61.4 (557)	66.3 (625)	
Secondary school	16.7 (152)	15.3 (144)	
College or university	21.9 (199)	18.4 (173)	0.299
**Family size**			
Mean ± SD	8.1 ± 2.4	8.9 ± 2.5	0.001
1-6 persons	27.6 (240)	12.5 (118)	
7-8 persons	34.7 (301)	37.6 (355)	
> 8 persons	37.7 (327)	49.9 (472)	< 0.001
**Household STL^b^**			
Mean ± SD	9.2 ± 2.9	8.0 ± 2.8	0.002
Low (0-6)	18.3 (156)	29.5 (292)	
Medium (7-10)	48.9 (418)	52.4 (518)	
High (11-16)	32.8 (280)	18.1 (179)	0.003
**Present household economic situation index^c^**			
Worse off (14-20)	17.9 (159)	30.7 (283)	
Medium (7-13)	56.5 (503)	54.1 (498)	
Better off (4-6)	25.6 (228)	15.2 (140)	< 0.001

SD: Standard deviation

^a^P for the comparison between governorates using χ^2 ^tests for categorical variables and t-tests for means.

^b^Household standard of living index (STL) was based on the possession of 16 household amenities; each item given a value of 1.

^c^Present household economic situation index was formulated based on 4 questions: - "Do you have enough money to cover family needs now?" "At the present time, do you have any debts?" At the present time, to what extent do you have to postpone paying bills?" and "During the past school year, did you have to borrow money to cover family needs?"

### Stunting

The prevalence of stunting was 7.3% in Ramallah and 6.5% in Hebron governorates. In both governorates, the prevalence of stunting was higher among boys than girls and this difference was more pronounced in Hebron (9.4% for boys versus 4.2% for girls, P < 0.001) (Table 2). Among boys in Ramallah, stunting was significantly higher among those with rural residence, low mother's education, low household STL, lack of food availability, and among those who had not reached puberty. In Hebron, stunting was significantly associated with worse off present household economic situation index (Table 3).

**Table 2:** Sample and population size weighted results of the anthropometric characteristics of the sample of 1942 Palestinian adolescents from Ramallah and Hebron governorates

	Ramallah				Hebron			
	
	Total^a^	Boys	Girls	P^b^	Total^a^	Boys	Girls	P^b^
	n = 935	n = 437	n = 498		n = 1007	n = 458	n = 549	
Age (years), mean (SE)	14.4 (0.1)	14.4 (0.1)	14.4 (0.1)	0.983	14.4 (0.1)	14.3 (0.1)	14.4 (0.1)	0.273
Age of onset of puberty (years), mean (SE)	13.1 (0.1)*	13.1 (0.1)	13.0 (0.1)	0.226	13.3 (0.1)*	13.5 (0.1)	13.1 (0.1)	0.015
Weight (kg), mean (SE)	53.4 (1.0)	53.5 (1.8)	53.3 (1.0)	0.899	51.1 (0.7)	50.1 (1.3)	51.9 (0.5)	0.206
BMI (kg/m^2^), mean (SE)	20.7 (0.3)	20.1 (0.4)	21.2 (0.3)	0.045	20.1 (0.2)	19.3 (0.3)	20.7 (0.2)	0.001
BMI percentile, mean (SE)	53.5 (2.1)	47.2 (2.4)	58.6 (2.4)	0.002	49.1 (1.8)	42.0 (2.5)	54.9 (1.5)	< 0.001
**BMI categories using the 2000 CDC reference**								
Underweight % (95% CI)	6.1 (4.4, 8.3)*	9.7 (7.7, 12.3)	3.1 (1.8, 5.3)		9.1 (7.1, 11.6)*	12.9 (9.5, 17.2)	6.0 (4.1, 8.7)	
Overweight % (95% CI)	12.9 (9.7, 16.9)	9.6 (7.2, 12.7)	15.6 (10.7, 22.2)	0.005	11.2 (9.1, 13.8)	8.5 (6.0, 12.0)	13.5 (10.5, 17.1)	0.001
Obesity % (95% CI)	7.0 (4.7, 10.4)	8.2 (4.1, 15.7)	6.0 (3.9, 9.3)		4.1 (2.9, 5.8)	4.9 (2.8, 8.5)	3.4 (2.2, 5.6)	
**BMI categories using the IOTF criteria**								
Overweight % (95% CI)	16.4 (12.9, 20.5)*	13.3 (10.6, 16.5)	18.9 (13.5, 25.9)		13.1 (10.9, 15.7)*	10.9 (7.9, 14.9)	14.9 (11.9, 18.6)	
Obesity % (95% CI)	4.1 (2.4, 7.1)	5.2 (2.0, 12.9)	3.3 (2.1, 4.9)	0.182	2.1 (1.3, 3.4)	2.2 (1.1, 4.6)	2.0 (1.0, 3.9)	0.230
Height (cm), mean (SE)	160.0 (0.7)	162.1 (1.1)	158.3 (0.5)	0.005	159.2 (0.4)	160.3 (0.8)	158.4 (0.3)	0.027
Height for age percentile, mean (SE)	38.1 (1.6)	36.9 (2.6)	39.1 (2.1)	0.510	36.1 (1.2)	33.3 (1.7)	38.5 (1.3)	0.022
Stunting % (95% CI)	7.3 (5.1, 10.4)	9.2 (5.2, 15.4)	5.9 (3.9, 8.8)	0.195	6.5 (5.1, 8.3)	9.4 (7.5, 11.7)	4.2 (2.8, 6.2)	< 0.001

SE: Standard error. BMI: body mass index, 95% CI: 95% confidence interval. CDC: Center for Disease Control and Prevention, IOTF: International Obesity Task Force.

*P < 0.05

^a^P is for the difference between total adolescents in Ramallah governorate and total adolescents in Hebron governorates using χ^2 ^for categorical variables and t-test for means.

^b^P is for the difference between genders in the same governorate using χ^2 ^for categorical variables and t-test for means.

Number of adolescents who have reached puberty: boys from Ramallah = 363, girls from Ramallah = 389, boys from Hebron = 365, and girls from Hebron = 465.

**Table 3:** Sample and population weighted results of stunting, underweight and overweight/obesity of Palestinian boys from Ramallah and Hebron governorates by sociodemographic characteristics (n = 895)

	Ramallah governorate				Hebron governorate			
	
	n	Stunting %	Underweight %	Overweight/ obese^a ^%	n	Stunting %	Underweight %	Overweight/ obese^a ^%
**Residence**								
Urban	197	4.8*	9.4	21.2	309	9.4	10.6	15.3*
Rural	228	13.1	9.6	15.8	133	10.6	18.9	6.8
**Age in years**								
13	180	7.2	9.4	21.1	181	7.2	12.2	11.1
14	192	9.4	12.1	15.0	210	10.5	12.9	13.3
15	65	10.7	5.2	18.8	67	11.9	14.8	19.4
**Mother's education**								
Low	307	10.8*	10.1	16.2	316	8.2	11.4	14.3
Medium/High	110	3.5	9.0	23.2	106	10.5	17.0	9.4
**Father's education**								
Low	270	11.6	10.2	14.4	278	9.7	13.2	13.8
Medium	64	5.1	10.2	32.4	59	13.6	6.8	13.6
High	84	5.0	9.0	17.6	84	2.4	15.6	10.7
**Family size**								
1-6 persons	129	5.4	6.6	25.6^e^	59	5.1	11.8	11.8
7-8 persons	142	10.8	11.3	20.0	166	8.4	10.2	16.9
> 8 persons	111	11.8	14.3	10.0	195	10.3	15.4	9.8
**Household STL^b^**								
Low	56	15.5^e^	7.0	9.2* ^e^	116	9.5	16.5	9.6
Medium	202	10.7	12.3	15.4	231	9.1	14.3	9.5
High	147	3.1	6.7	25.1	103	7.8	4.8	25.3***
**Present household economic situation index^c^**								
Worse off	68	8.8	11.0	12.8	120	13.4*	15.8	15.2
Medium	233	11.5	10.9	14.8	224	8.5	12.5	10.3
Better off	115	5.8	6.6	26.5	73	2.8	9.6	17.8
**Food availability^d^**								
Not available	128	13.5**	13.2	11.7	193	10.9	14.5	11.5
Available	292	7.5	8.8	20.6	229	7.0	11.3	14.4
**Onset of puberty**								
No	74	16.6*	14.2	17.2	92	15.2	25.0	7.6**
Yes	363	7.8	9.0	17.9	365	8.0	9.9	14.6

*P < 0.05 **P < 0.01 ***P < 0.001

^e^*P *for trend < 0.05

^a^Overweight/obesity were defined using the 2000 CDC reference.

^b^Household standard of living index (STL) was based on the possession of 16 household amenities; each item given a value of 1.

^c^Present household economic situation index was formulated based on 4 questions: - "Do you have enough money to cover family

needs now?" "At the present time, do you have any debts?" At the present time, to what extent do you have to postpone paying bills?" and "During the past school year, did you have to borrow money to cover family needs?".

^d^Food availability was based on the question: is there enough food for all family members on daily basis now?

Among girls, the proportion being stunted was significantly higher among those from rural areas of Ramallah, among those who had not reached puberty in both governorates and among girls with low STL in Hebron (Table 4).

**Table 4:** Sample and population weighted results of stunting, underweight and overweight/obesity of Palestinian girls from Ramallah and Hebron governorates by sociodemographic characteristics (n = 1047)

	Ramallah governorate				Hebron governorate			
	
	n	Stunting %	Underweight %	Overweight/ obese^a ^%	n	Stunting %	Underweight %	Overweight/ obese^a ^%
**Residence**								
Urban	176	1.9**	3.3	24.0	354	3.4	6.8	18.6
Rural	309	7.9	3.1	20.7	182	6.0	5.0	14.2
**Age in years**								
13	207	5.6	1.9	22.1	150	6.0^e^	7.4	20.7
14	224	6.3	4.7	21.9	274	4.7	4.4	16.1
15	67	5.2	1.0	20.2	125	1.0	8.0	14.4
**Mother's education**								
Low	355	6.0	2.6	22.2	413	5.1	6.3	16.4
Medium/High	139	5.2	4.6	19.4	109	0.9	6.5	17.4
**Father's education**								
Low	287	5.7	3.5	23.8	347	4.6	6.3	17.8
Medium	88	6.5	1.7	15.7	85	4.7	7.0	13.0
High	115	5.9	3.1	19.2	89	2.2	5.6	14.6
**Family size**								
1-6 persons	111	2.8	1.4	29.1^e^	59	5.1	5.1	18.4
7-8 persons	159	6.1	3.6	24.7	189	4.8	6.4	17.5
> 8 persons	216	7.0	3.5	16.9	277	3.6	6.5	15.5
**Household STL^b^**								
Low	100	8.4	4.0	12.7^e^	176	7.9**	5.1	14.8
Medium	216	6.4	2.5	21.8	287	2.4	6.6	18.1
High	133	3.6	3.3	26.2	76	2.7	6.6	18.3
**Present household economic situation index^c^**								
Worse off	91	5.2	1.8	26.1	163	3.6	8.0	15.4
Medium	270	6.2	3.4	20.5	274	5.1	5.5	16.0
Better off	113	5.7	4.0	16.8	67	3.0	7.5	17.9
**Food availability^d^**								
Not available	165	4.0	2.7	26.6	255	4.7	7.1	15.7
Available	327	6.8	3.3	19.0	269	3.7	5.6	17.4
**Onset of puberty**								
No	100	15.6**	6.4	11.5**	84	10.6**	20.3	3.7***
Yes	389	4.2	2.5	23.5	465	3.0	3.5	19.3

*P < 0.05 **P < 0.01 *** *P *< 0.001

^e^*P *for trend < 0.05

^**a**^Overweight/obesity were defined using the 2000 CDC reference.

^b^Household standard of living index (STL) was based on the possession of 16 household amenities; each item given a value of 1.

^c^Present household economic situation index was formulated d based on 4 questions: - "Do you have enough money to cover family needs now?" "At the present time, do you have any debts?" At the present time, to what extent do you have to postpone paying bills?" and "During the past school year, did you have to borrow money to cover family needs?".

^d^Food availability was based on the question: is there enough food for all family members on daily basis now?

Results from the multivariate regression analysis (Table 5) in boys showed that high father's education was negatively associated with stunting and that stunting was higher among boys in the 15 years age group. Among girls, stunting was negatively associated with urban residence, with medium STL and it was lower among girls who have reached puberty. No colinearity or significant interactions were detected between variables in the models.

**Table 5:** Sample and population weighted results of the multivariate logistic regression models for the relationship between stunting, overweight/obesity and selected sociodemographic variables in a sample of 1942 Palestinian adolescents in Ramallah and Hebron governorates

	Boys				Girls			
	
	Stunting		Overweight/obesity^a^		Stunting		Overweight/obesity^a^	
	
	OR	95% CI	OR	95% CI	OR	95% CI	OR	95% CI
**Region**								
Hebron	1		1		1		1	
Ramallah	1.37	0.75-2.51	1.57	0.93-2.65	1.26	0.76-2.09	1.38	0.87-2.18
**Residence**								
Rural	1		1		1			
Urban	0.96	0.51-1.81	1.27	0.68-2.38	0.45	0.23-0.89*		
**Age in years**								
13	1		1		1		1	
14	1.45	0.83-2.51	0.93	0.57-1.54	0.94	0.55-1.60	0.75	0.46-1.23
15	2.15	1.16-3.98*	1.58	0.91-2.74	0.37	0.13-1.06	0.61	0.33-1.10
**Father's education**								
Low	1							
Medium	1.18	0.51-2.72						
High	0.23	0.07-0.74*						
**Mother's education**								
Low	1							
Medium/high	1.91	0.89-4.09						
**Family size**								
1-6 persons	0.57	0.23-1.40	1.78	0.97-3.28			1.42	0.82-2.47
7-8 persons	1.06	0.56-2.04	1.77	0.96-3.24			1.24	0.80-1.93
> 8 persons	1		1				1	
**Household STL^b^**								
Low	1		1		1			
Medium	0.82	0.37-1.79	1.10	0.64-1.88	0.45	0.25-0.82*		
High	0.94	0.35-2.53	2.08	1.19-3.63*	0.43	0.13-1.39		
**Present household economic situation index^c^**								
Worse off	1		1					
Medium	0.85	0.45-1.62	0.64	0.34-1.22				
Better off	0.28	0.08-1.02	0.97	0.53-1.77				
**Food availability^d^**								
Not available	1							
Available	0.88	0.56-1.38						
**Onset of puberty**								
No	1		1		1		1	
Yes	0.50	0.25-1.02	1.53	0.74-3.19	0.26	0.13-0.49***	4.80	2.29-10.04***

OR: Odds ratios, 95%CI: 95% confidence intervals

*P < 0.05 **P < 0.01 ***P < 0.001

^**a**^Overweight/obesity were defined using the 2000 CDC reference.

^b^Household standard of living index (STL) was based on the possession of 16 household amenities; each item given a value of 1.

^c^Present household economic situation index was formed based on 4 questions: - "Do you have enough money to cover family needs now?" "At the present time, do you have any debts?" At the present time, to what extent do you have to postpone paying bills?" and "During the past school year, did you have to borrow money to cover family needs?"

^d^Food availability was based on the question: is there enough food for all family members on daily basis now?

Stunting was positively associated with underweight. Of the stunted adolescents, 20.8% were underweight compared to 7.2% of the normal adolescents (p < 0.001), while 71.5% had normal weight and 7.7% were overweight/obese (data not shown).

### Underweight, overweight and obesity

The prevalence of underweight among adolescents was 6.1% in Ramallah and 9.1% in Hebron. In both governorates, the proportion being underweight was significantly higher among boys than girls (9.7% among boys versus 3.1% among girls in Ramallah and 12.9% versus 6.0%, respectively in Hebron, (P < 0.01) (Table 2).

The prevalence of overweight and obesity was higher in Ramallah than Hebron. Using the 2000 CDC reference, the prevalence of overweight for both genders combined was 12.9% in Ramallah and 11.2% in Hebron, while the corresponding figures for obesity were 7.0% and 4.1%, respectively. Using the IOTF criteria, the prevalence of overweight was 16.4% in Ramallah and 13.1% in Hebron, and the corresponding figures for obesity were 4.1% and 2.1%, respectively. Girls were found to be heavier than boys in both governorates. The mean BMI percentile for girls was well above the 50^th ^percentile of the reference population (58.6 in Ramallah and 54.9 in Hebron); while among boys the mean BMI percentile was below (47.2 in Ramallah and 42.0 in Hebron) (Table 2).

Tables (3, 4) present the factors associated with overweight/obesity among boys and girls as defined by the 2000 CDC reference. Among boys in Ramallah, overweight/obesity was significantly higher among those with high STL (25.1%) versus those with medium (15.4%) and low STL (9.2%). A significant positive trend of increase in overweight/obesity was noted with decrease in family size. Among boys in Hebron, overweight/obesity was significantly associated with urban residence, high household STL and was significantly higher among those who have reached puberty.

Among girls, a significant positive trend of increase in overweight/obesity was noted with decrease in family size and increase in household STL in Ramallah but not in Hebron. In both governorates, girls who had reached puberty had significantly higher prevalence of overweight/obesity than those who have not yet reached puberty.

Results from the multivariate regression analysis (Table 5) showed that high STL was independently associated with overweight/obesity in boys; while in girls only onset of puberty was significantly associated with overweight/obesity. No colinearity or significant interactions were detected between variables in the models. Results from multivariate regression analysis for underweight showed that in boys, underweight was negatively associated with high STL and onset of puberty; while in girls it was negatively associated with residence in Ramallah governorate and onset of puberty (data not shown).

## Discussion

Results of selected height and weight measurements for assessing nutritional status and their sociodemographic covariates in a sample of Palestinian adolescents aged 13-15 years from the two major West Bank governorates, Ramallah and Hebron, are reported. The findings revealed that conditions related both to overnutrition and undernutrition are present in these two governorates. Higher prevalence of overweight/obesity was found among Ramallah adolescents compared to their counterparts in Hebron and a higher prevalence among girls than boys in both governorates is reported. The prevalence rates of underweight and stunting were higher among boys than among girls in both governorates.

Girls were slightly over-represented in the sample because there were slightly more girls than boys in these two grades in schools. Most of the students were in the 13 and the 14 years age groups because these are the most common age groups attending the 8^th ^and the 9^th ^grades. Fewer of the 9^th ^grade students, and less frequently 8^th ^grade students, would be in the 15 years age group. The proportion of the younger age group (age group 13) was slightly higher and that of the older age groups (14 and 15 years age groups) was slightly lower in Ramallah than Hebron because the proportion of 8^th ^grade students in Ramallah governorate was higher than that in Hebron governorate.

In our study, indicators of both present and past family wealth were used. Closures of Palestinian areas, and recurrent curfews since the second Intifada in 2000 have led to a rise in poverty [20] with 34% of Palestinians reported to be food insecure in 2006 [21]. The STL index, reflecting past wealth, is amenities-based and is similar to what has been used in other surveys on adolescents [17,22]. It is thought to be useful in this age group as many are unable to provide substantive information on their parents' occupation and income [23]. The amenities included in this index are culture specific for Palestinians and are listed among those used by PCBS for assessment of material standards of living [24]. In contrast, the present household economic situation index, reflecting recent deterioration in wealth, was based on reports of current economic hardships and financial coping mechanisms. The weak correlation between STL and the present household economic situation index (0.42) suggests that they reflect different aspects of family affluence.

Affluence and urban lifestyle are associated with a higher prevalence of overweight in lower and middle income developing countries [7]. This was the case in this study where adolescents in Ramallah governorate had a higher prevalence of overweight/obesity than in Hebron; probably due to higher standards of living [14]. Adolescents in Ramallah had better indicators of economic situation such as STL index and the present household economic situation index. They also had better social indicators such as smaller family size. Furthermore, the prevalence of overweight/obesity was higher in urban areas and among those with high STL and this was more pronounced among boys. Similar associations of overweight among boys with high family affluence scale were found in the Palestinian HBSC study [12].

Overweight/obesity was higher among girls than boys. In Palestine, boys are reported to be more physically active than girls [25]. Cultural restrictions on girls imply that they spend more time indoors and thus have easier access to food. These findings are similar to those found in a 2002 North Gaza Strip study, in which 15.4% of the boys and 20.2% of the girls aged 12-15 years were found to be overweight/obese (using the 2000 CDC reference) based on measured weights and heights [16]. However, the validity of this comparison may be questionable now as the economic situation has deteriorated considerably in Gaza since 2002 [20]. An opposite trend in gender difference was found in the Palestinian HBSC survey conducted among adolescents aged 12-18 years in 2003/2004 in the West Bank and the Gaza Strip. This survey reported overweight/obesity (using IOTF criteria) of 20.4% among boys and 13.0% among girls [12], while in our study the corresponding percentages by the same criteria were higher in girls (22.2% in Ramallah and 16.9% in Hebron) than in boys (18.5% in Ramallah and 13.1% in Hebron). One explanation might be that the Palestinian HBSC study used self-reported weights and heights. Self-reported data tend to underestimate overweight prevalence, especially among girls [26]. Moreover, the age groups were different between the HBSC study and our study, and the sample in our study was selected from only two governorates in the West Bank while the HBSC study was a national-level study in the West Bank and the Gaza Strip. In the Palestinian HBSC study, 27.5% of the data was missing for BMI. In contrast, there was no missing data in our study.

A 2003-2004 national survey of Jewish and non-Jewish Israeli school adolescents (aged 11-19 years) found that among boys, the prevalence of overweight/obesity (using the 2000 CDC reference), based on measured heights and weights, was 19.6% for Jews and 22.6% for non-Jews (mostly Arabs and Druze) while in girls it was 15.7% for Jews and 20.3% for non-Jews [27]. Also in this case, the gender difference was opposite to the findings in our study. Such a comparison should also be taken with caution because of a much wider age range and sampling frame in this study compared to our more limited study. In Lebanon, boys in private schools (aged 12.5-15 years) in 2002-2003 were found to have higher rates of overweight and obesity than girls using measured weights and heights. The prevalence of overweight and obesity (using IOTF criteria) among boys was 29.2% and 8.7%, respectively. The corresponding figures for girls were 18.7% and 3.7%, respectively. Western feminine self-image and fears of obesity were more marked among Lebanese girls than boys [28].

Studies in the Gulf areas, enjoying higher standards of living than the West Bank, showed a higher prevalence of overweight/obesity than our figures. Some of these studies showed higher prevalence of overweight/obesity among boys and other studies showed higher prevalence among girls. A study in 2003-2004 among Qatari adolescents aged 12-17 years found a prevalence of overweight and obesity based on measured weights and heights (using IOTF criteria) of 28.6% and 7.9% among boys, respectively and 18.9% and 4.7% among girls, respectively [29]. In contrast, studies in 2000 in Bahrain among adolescents aged 12-17 years found a higher rate of overweight and obesity among girls based on measured weights and heights. The prevalence of overweight and obesity among boys (using IOTF criteria) were 15.3% and 14.9%, respectively while the corresponding figures for girls were 24.5% and 17.9%, respectively [30].

As reported in other studies [22], overweight/obesity was associated with family size. The mean family size in the West Bank is 5.5 [11]; however, it was larger in our study (8.1 in Ramallah and 8.9 in Hebron) as the sample represents a selected segment of families that have progressed further into their life cycle.

The prevalence rates of underweight were lower in Ramallah than in Hebron. Multivariate regression analysis showed that underweight was negatively associated with residence in Ramallah among girls and with high STL among boys. Stunting rates were similar in both governorates. Both underweight and stunting were higher among boys than among girls in both governorates. The same pattern between boys and girls of higher underweight (7.0% versus 3.8%) and higher stunting (13.7% versus 6.2%) among boys was reported in the 2002 Gaza study [16]. Similar patterns of higher underweight [29] and higher stunting among boys [31,32] have been also found in other developing countries. Higher stunting among Palestinian boys might indicate that they suffer from micronutrient deficiencies as they are prone to make less healthy food choices [25], or it could be due to age-specific differences in catchup growth between the sexes [33]. Stunting might also be associated with parasitic infections as suggested by some studies in developing countries [34]. A recent study from Gaza strip showed that the prevalence of intestinal parasites was 34.2% in children aged 6-11 years [35]. Since we neither could find recent data nor collected such data in the West Bank, we may only speculate if the higher prevalence of stunting in boys could be due to higher risk of infections as boys spend more time outdoors than girls. The prevalence of stunting among girls was low in general, but it was relatively high among girls living in rural areas and among those with low STL. Short stature may have an impact on girls and their reproductive outcomes; this may be even more important in the Palestinian context given the young median age of first marriage of 18 years and the high total fertility rate of 4.6 [10]. Onset of puberty was positively associated with overweight/obesity and negatively associated with stunting and underweight. This might be explained by a larger proportion of the well nourished amongst those who have reached puberty in the sample. Similar association of overweight/obesity with onset of puberty has been found in other studies [16,22]. Although asking about Tanner stage would have given a more precise measure of sexual maturation, cultural taboos did not permit this.

## Limitations

The study had certain limitations such as not including adolescents out of schools. The sample was not a national sample which makes it difficult to generalize the results to the whole of the oPt and limits the opportunity to compare our findings with similar data from other countries in the region. The sample was based on 8^th ^and 9^th ^grade students and the results reflect the prevalence of anthropometric measures in these two grades only. The corresponding age groups were mainly 13 and 14 years old, while the age group of 15 years was relatively small. Weights and heights were taken only once by one field worker, so that some measurements errors might not be accounted for.

The student's questionnaire was not validated, but some questions were taken from the validated HBSC questionnaire [17] and field workers were present when administering the questionnaire to explain questions when needed. A pilot study was done and unclear questions were modified. Moreover, the questionnaire was tested for reliability (a 1-week test-retest). The measures for the onset of puberty and household amenities had very high agreement between test and retest. The 1 week test-retest for the onset of puberty showed consistent answers in 92.3% in boys and 95.2% in girls, and that of the ownership of household amenities showed consistent answers ranging between 86.1% and 99.1%. Other strengths of the study include the fact that actual measurements of the weights and the heights were taken, its relatively large sample size, single stage cluster sampling method, the urban/rural distribution, and the high response rate. We also studied the association with certain sociodemographic characteristics to identify the vulnerable groups for malnutrition.

## Conclusion

Our findings suggest that both overnutrition and undernutrition coexist in the study areas. This stage of nutrition transition is similar to low income economies where both under- and overnutrition coexist [36] and where overnutrition is more prevalent among the more affluent strata of the society [7]. Both conditions may predispose to chronic diseases later in life. Urbanization, poverty, limited mobility and cultural restrictions on girls may all play a role.

Future studies should address causes of overnutrition and undernutrition among Palestinian adolescents with relevance to preventive measures that could be instituted by the Palestinian health system given its limited resources. There is urgent need to improve nutrition to combat stunting, and to raise awareness about overweight and its associated health risks.

## Competing interests

The authors declare that they have no competing interests.

## Authors' contributions

NM contributed to the conception, design, data collection, analysis and interpretation of data, and drafted the manuscript; HFA and GHO contributed to the conception, design, analysis and interpretation of data, and revising the manuscript. FA contributed to sample design, analysis and interpretation of data. All authors gave approval of the final version.

## Pre-publication history

The pre-publication history for this paper can be accessed here:

http://www.biomedcentral.com/1471-2458/9/485/prepub
